# What is hiding in the hindgut sac? Looking beyond rectal carcinoma

**DOI:** 10.1007/s13244-014-0347-z

**Published:** 2014-07-21

**Authors:** Vivek Virmani, Subramaniyan Ramanathan, Vineeta Sethi Virmani, John Ryan, Najla Fasih

**Affiliations:** Department of Diagnostic Imaging, The Ottawa Hospital, General campus, University of Ottawa, 501 Smyth road, Ottawa, ON K1S8L6 Canada

**Keywords:** Anorectal, Magnetic resonance, Computed tomography, Neoplasms, Cross-sectional

## Abstract

**Objectives:**

Although rectal cancer is by far and large the most common pathology involving the rectum that needs imaging, there are many other important but less common pathological conditions affecting anorectal region. The objective of this pictorial review is to discuss the cross-sectional imaging features of less common anorectal and perirectal diseases.

**Results:**

Although a specific histological diagnosis cannot usually be made due to considerable overlap in the imaging appearances of anorectal diseases, this review illustrates the cross-sectional imaging findings with emphasis on magnetic resonance imaging (MRI) that can help in narrowing down the differentials to a reasonable extent.

**Teaching points:**

• *Variety of pathology exists in the anorectum apart from common rectal carcinoma*

• *Anorectal diseases present as non-specific wall thickening indistinguishable from rectal carcinoma*

• *Computed tomography (CT) and MRI can help in narrowing down the differentials, although often biopsy is warranted*.

## Introduction

Primary rectal adenocarcinoma is a common malignancy with a high mortality rate in the western world. Initially computed tomography (CT) and endoluminal ultrasound (EUS) have been the mainstay of diagnosis and staging. In the past decade magnetic resonance imaging (MRI) has become the imaging modality of choice for loco-regional staging of rectal cancer. The complex anatomy of the anorectal region makes imaging and interpretation challenging. The advent of MRI, with its high soft tissue contrast, multiplanar capability and no radiation risk, has simplified imaging of the anorectal region. The role of MRI in preoperative staging of rectal cancer has been well established now. Though rectal cancer is the commonest cause of rectal mass, there are many other common and uncommon diseases which affect rectum and perirectal region, many of which can mimic rectal carcinoma and their distinction is essential as the management strategy changes significantly [1]. The objective of this review is to describe and illustrate the specific imaging findings of uncommon and atypical diseases affecting the anorectal region. Although a specific diagnosis cannot usually be made due to considerable overlap in the imaging appearances of anorectal diseases, this review illustrates the cross-sectional imaging findings with emphasis on MRI that can help to narrow down the differential diagnoses to a reasonable extent. Uncommon anorectal diseases include congenital cysts, benign and malignant neoplasms excluding adenocarcinoma, atypical infections, inflammatory conditions (excluding common inflammatory bowel disease and post radiation changes) and a few other rare miscellaneous conditions.

## MR imaging technique

MRI of the rectum may be performed with either an endorectal coil or a phased-array surface coil. The decision depends on the availability of coils, technical expertise of radiologists, surgeon’s preference and practical issues like time constraints. In our institution we use phased array surface coils routinely for all rectal diseases. The advantage of an endorectal coil lies in its high-resolution images that fully depict the wall layers of the bowel but has the disadvantage in evaluating rectal strictures, high rectal carcinomas and assessment of the perirectal structures due to its smaller field of view (FOV). Phased-array surface coil yields high-spatial-resolution images, albeit with less distinction of bowel wall layers but with additional advantage of a large field of view, patient comfort and ease of use in structuring cancers and high rectal, rectosigmoid tumours [2, 3].

In our institution we do no routine bowel preparation, rectal contrast or antispasmodic agents. Opacification rectal lumen with contrast is still controversial with use of various agents like super paramagnetic iron oxide solutions, methylcellulose, barium suspensions and aqueous gel [4].

A sagittal T2-weighted turbo spin-echo (TSE) sequence is obtained first to locate the rectal lesion. Based on the sagittal sequence, axial and coronal T2-weighted TSE sequences are planned, and they are angled to the plane exactly perpendicular and parallel to the lesion. We have a standard rectal cancer protocol which is applied for all other rectal lesions as well [2]. Our MRI protocol consists of sagittal T2-weighted single-shot images and T2-weighted TSE images in the axial and coronal planes. High-resolution images are obtained in the axial and coronal planes with a slice thickness of 3 mm and small FOV. Unenhanced and contrast-enhanced axial and coronal high-resolution T1-weighted fat-saturated images of the rectum are also obtained. The use of gadolinium in rectal cancers is now becoming optional, as all the staging parameters are adequately depicted in T2-weighted sequences. However, rectal lesions other than usual cancers still benefit from gadolinium. As of now, we routinely do post gadolinium multiplanar T1 VIBE (volume interpolated breath-hold examination) sequences.

Diffusion-weighted imaging (DWI) is a functional imaging tool that yields information about water mobility and tissue cellularity. It also allows calculation of the apparent diffusion coefficient (ADC) from images with different *b* values. Although diffusion sequences are not part of our routine rectal MRI protocol, there is increasing literature on their usefulness, especially for malignant lesions. The reader is requested to refer to various articles available on principles, imaging parameters and pitfalls of diffusion imaging, as these are beyond the scope of our article [5, 6]. Malignant tumours are generally depicted as foci of increased intensity on DWI and decreased signal intensity on ADC images, because water diffusion is restricted in highly cellular tissues in malignant tumours [5, 7, 8]. However, blood, fat, abscesses, lymph nodes, and melanin can show restricted diffusion and can be resolved by referring to standard T1- and T2-weighted images [9]. Examples include dermoid cyst, rectal lipoma, melanoma and endometriosis. DWI has been proven useful in diagnosis, assessing treatment response and recurrence of rectal carcinoma [10–12]. Other malignant lesions that can show diffusion restriction include lymphoma, stromal tumours and mucinous carcinoma [10, 13–15]. Data on other rectal tumours are not well established and mostly are extrapolated from similar tumours occurring at other sites. Cystic lesions (duplication cyst, tail gut cyst, pseudomyxoma) show T2 shine-through effect, which is high signal intensity on low- and high-*b*-value images and on ADC images [9].

## Developmental retro-rectal cysts

Developmental cysts are benign epithelial cysts in the retro-rectal space, arising from caudal embryonic vestiges. Often these are incidentally detected in middle-aged women. Developmentally these are sub-typed in to epidermoid cysts, dermoid cysts, enteric cysts and neuroenteric cysts. These cysts, when large, can create a mass effect on the rectum and can indent the rectum mimicking sub-mucosal mass.

**Fig. 1:**
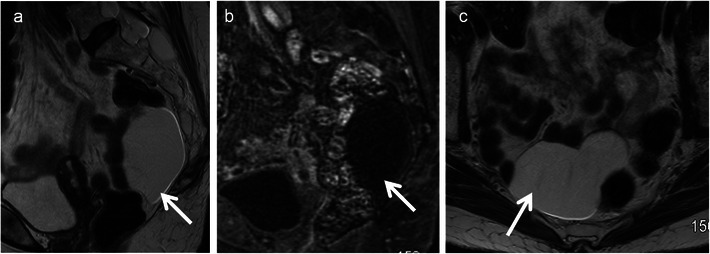
Retrorectal or tailgut cyst in a 42-year-old woman. **a** Sagittal T2-weighted MRI reveals unilocular cystic lesion (*arrow*) in the retrorectal location. **b** Sagittal T1-weighted MRI image reveals the lesion (*arrow*) to be hypointense. **c** Axial T2-weighted MRI again demonstrates the cystic lesion (*arrow*)

Enteric cysts are most frequent among these and are lined with enteric mucosa. It includes retro-rectal cystic hamartoma (RRCH), also known as tail gut cyst and rectal duplication cyst. Enteric cysts typically are unilocular or multilocular lesions that are hypointense on T1 and hyperintense on T2 without internal enhancement (Fig. 1). RRCH is often multiloculated and may have a small peripheral cyst with honeycomb appearance, which is an important differentiating feature from the other types of developmental cysts. Rectal duplication cysts are different from RRCH in that they have muscular layer and the only lesion in contiguity or continuity with the rectum. They can be associated with genitourinary anomalies.

Neuroenteric cysts are differentiated from other cysts only on histology and are indistinguishable on imaging. Epidermoid and dermoid cysts are lined with stratified squamous epithelium and they have classical features as in any other body location. Epidermoid cysts are unilocular with thin wall and clear fluid. Tiny discrete and linear T2 hypointense areas suggesting keratin and diffusion restriction is characteristic of epidermoid cyst [16, 17]. Dermoid cysts contain fat with skin appendages, such as tooth buds, sweat glands and hair follicles. Fat suppression MRI is helpful in confirming the dermoid cyst and can show diffusion restriction [9].

Role of MRI is to locate the cysts in relation to the rectal wall, its continuity with rectal lumen and assess complications [18, 19]. Complications of developmental cysts include infection, haemorrhage, fistula formation and malignant degeneration. High signal on T1-weighted images can be due to mucus, proteinaceous material, haemorrhage or fat. Low-signal areas on T2-weighted images can be due to haemorrhage and aggregates of keratin. Mural nodularity, asymmetric wall thickening and enhancement suggest malignant change.

## Neoplasms

Primary adenocarcinoma is the commonest rectal neoplasm, constituting 90–95 % of rectal cancers. Other less common tumours include benign neoplasms like lipoma, leiomyoma, schwannoma, villous adenoma, cavernous haemangioma and malignant neoplasms like lymphoma, gastrointestinal stromal tumours (GIST), carcinoid, melanoma, Kaposi sarcoma and secondary neoplastic involvement [3].

## Benign neoplasms

### Lipoma

Rectal lipoma is an uncommon benign submucosal tumour arising from deposits of adipose connective tissue in the bowel wall. Almost 70 % of gastrointestinal lipomas are located in the right colon, and their frequency gradually decreases from cecum to rectum [20]. The most common symptoms are abdominal pain, bleeding, signs of obstruction, signs of intussusception.

Most lipoma present as a broad-based mass with a smooth, well-demarcated margin. Although submucosal in origin, lipoma occasionally evolves into pedunculated lesions. CT shows typical fat attenuation mass in the bowel wall [21]. MRI readily demonstrates the fatty nature of the mass as being isointense compared with the subcutaneous fat on T1-weighted imaging, and low signal intensity on T1-weighted imaging with fat suppression, which confirms the diagnosis of lipoma. Contrast enhancement is variable, usually mild [22].

### Leiomyoma

Leiomyoma is a benign smooth muscle tumour and differs clinically, histologically and immunophenotypically from gastrointestinal stromal tumours (GISTs). Leiomyoma shows positivity for smooth muscle actin and desmin, and they are not kit-positive in contrast to that of GISTs. Exact incidence is not known as only few scattered case reports are available. It rarely occurs in the rectum. Appearance is non-specific on CT with localised intramural soft tissue mass showing mild to variable contrast enhancement. Cross-sectional imaging may demonstrate an exoenteric component in some cases. The MRI findings of rectal leiomyomas have not been well described, but it is reported that they are isointense to mildly hyperintense on T2-weighted images when compared with that of muscle [1].

### Villous adenoma

**Fig. 2:**
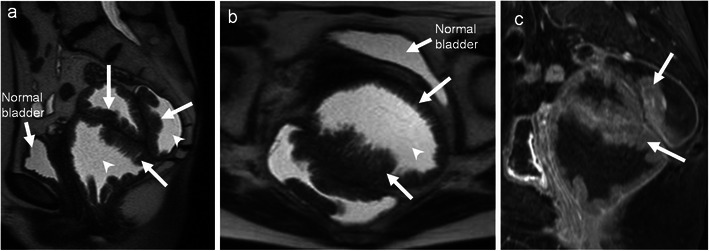
A 61 year-old-man presenting to the emergency department with severe dehydration resulting from intermittent episodes of diarrhoea and profuse mucous discharge from the rectum, found to have villous adenoma. **a** Sagittal T2-weighted MRI and **b** axial T2-weighted MRI reveal a carpet-like “hairy” lesion covering the mucosa of the rectum (*arrows*). The lumen of the rectum is distended and fluid filled (*arrowhead*) with no proximal obstruction. **c** Contrast-enhanced MRI reveals enhancement of the thickened abnormal mucosa (*arrows*)

**Fig. 3:**
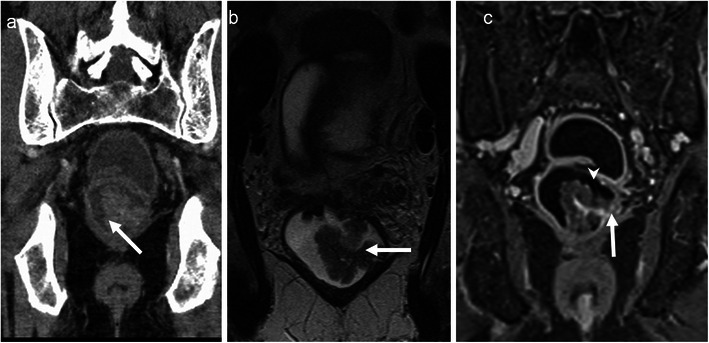
A 65-year-old woman presenting with intermittent diarrhoea, rectal bleeding and difficulty in defecation for 1 month. Laboratory data revealed hypokalaemia. **a** Coronal contrast CT reveals a lobulated solid mass (*arrow*) projecting into the lumen of the rectum. **b** Coronal T2-weighted MRI reveals the frond-like morphology of the lesion (*arrow*). **c** Coronal contrast MRI depicts a central vascular stalk (*arrow*) and the cauliflower or frond-like morphology (*arrowhead*), classical for villous adenoma

Adenomas of the colon are divided into three subtypes: tubular, villous and tubulo-villous. Villous adenoma constitutes approximately 10 % of all adenomas of the colon and is frequently encountered in the rectum and the sigmoid of elderly patients. Malignant potential of adenomatous polyps increases with size, villous configuration and degree of dysplasia. Patient often present with rectal bleeding, electrolyte imbalance (secrete copious amounts of mucoid material rich in protein and potassium) or difficulty in defecation. On imaging villous adenoma are frequently large with flat carpet like or cauliflower/frond like appearance (Figs. 2 and 3). Central vascular stalk with secondary branches is characteristic [23]. Surgical treatment is indicated as villous tumours have a 10–40 % chance of malignant transformation.

### Schwannoma

**Fig. 4:**
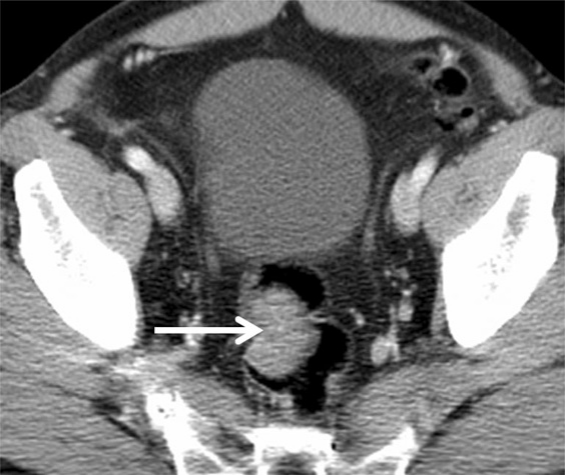
Axial contrast-enhanced CT shows histopathology proven rectal schwannoma presenting as non-specific homogeneous submucosal mass (*arrow*)

Rectal schwannomas are uncommon, the common sites in the gastrointestinal tract being oesophagus, stomach and small intestine in that order. Imaging findings are indistinguishable from other benign submucosal tumours like GIST and leiomyoma. On cross-sectional imaging they appear as encapsulated homogenous masses showing mild contrast enhancement (Fig. 4) [24]. Benign schwannomas were often misdiagnosed as leiomyomas, or leiomyosarcomas without immunohistochemical studies.

### Diffuse cavernous haemangioma

**Fig. 5:**
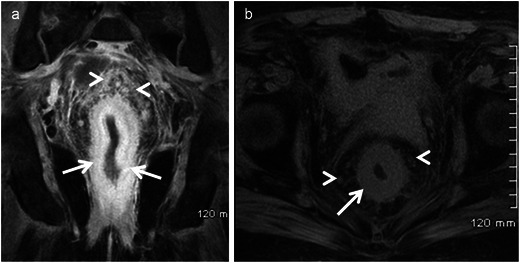
Diffuse cavernous haemangioma of the rectum in a 21-year-old man presenting with painless rectal bleeding. **a** Coronal and **b** axial fat-suppressed contrast-enhanced T1-weighted MRI reveal enhancing concentric rectal wall thickening (*arrows*) with enhancing serpiginious structures (*arrowhead*) in the mesorectum

Rare benign vascular malformation or hamartoma and is not a true neoplasm. The classical clinical triad of hematochezia, cutaneous haemangioma and ectopic pelvic phleboliths on radiograph should make one suspicious of intestinal haemangioma before the advent of cross-sectional imaging. Markedly thickened rectosigmoid wall showing moderately high T2-weighted signal intensity with high signal heterogeneous perirectal tissue with enhancing serpiginious structures (small vessels supplying the malformation) are the characteristic MRI findings (Fig. 5) [25, 26]. MRI may also help in identifying the extent of involvement and for possible extension in to bladder or even sacrum.

## Malignant neoplasms

### Uncommon or atypical rectal carcinoma

**Fig. 6:**
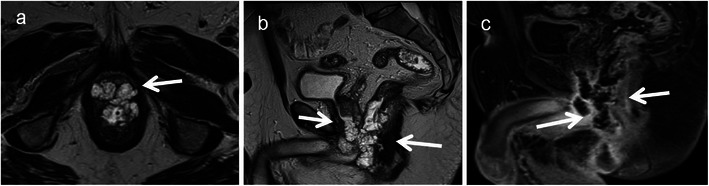
Mucinous (colloid) carcinoma of the rectum. **a** Axial and **b** sagittal T2-weighted MRI show intensely hyperintense heterogeneous rectal mass infiltrating the adjacent structures (T4 stage). (**c**) Sagittal fat-suppressed contrast-enhanced T1-weighted MRI reveal mesh-like peripheral enhancement of the rectal mass

Mucinous (colloid) and signet ring are two uncommon histological subtypes of rectal cancer with different biological behaviour and imaging appearances. Mucinous or colloid carcinoma is characterised by the production of an abundant amount of extracellular mucin. It has greater tendency for metastases and local recurrence with 5-year survival rate of 11 % compared with 57 % for non-mucinous carcinomas. Typical MRI features include high signal intensity on T2-weighted images with higher tumour-to-muscle, tumour-to-fat and tumour-to-urine signal intensity ratios. The solid component of the tumour can show diffusion restriction. Contrast-enhancement pattern is peripheral or heterogeneous with lace-like peripheral enhancement being characteristic (Fig. 6) [27].

Signet ring carcinoma is a rare type of rectal carcinoma, similar to the mucinous type in having a mucinous component, but with more aggressive behaviour in the form of a more advanced stage of initial diagnosis, more lymphatic and local spread and poor response to surgery. The most common imaging features are long segment of concentric wall thickening and a target appearance similar to those of metastatic linitis plastica consisting of thickened inner (mucosa and submucosa) and outer (serosa) layers and a relatively thin hypoattenuated middle layer (muscularis propria), or bowel wall thickening with homogeneous attenuation [28–30].

### Lymphoma

**Fig. 7:**
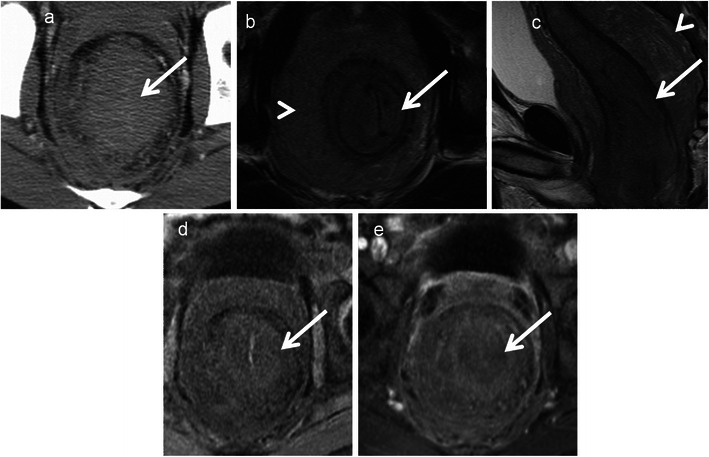
Burkitt cell lymphoma of the rectum in a 16-year-old male. **a** Axial CT shows Bulky tumour (*arrow*) infiltrating the rectum with luminal compromise. **b** Axial and **c** sagittal T2-weighted MRI reveal infiltration of the submucosal and muscular layers of the rectum with the tumour (*arrow*). The mucosal architecture and the shape of the rectum are maintained. The tumour is homogeneously isointense to hypointense on T2-weighted MRI. There is extensive perirectal infiltration (*arrowhead*). **d** Axial T1-weighted MRI reveals the isointense tumour (*arrow*) and **e** contrast-enhanced MRI reveals mild enhancement (*arrow*)

**Fig. 8:**
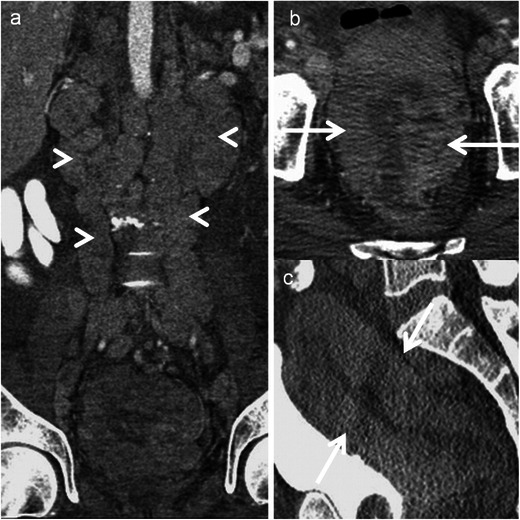
Mantle cell lymphoma in a 58-year-old man presenting with rectal bleeding and tenesmus.**a** Coronal, **b** axial and **c** sagittal contrast-enhanced CT reveal a long segment of marked circumferential wall thickening (*arrow*) involving the rectum with multiple polypoidal intraluminal projections. There is aneurismal dilatation of the rectum with extensive retroperitoneal lymphadenopathy (*arrowhead*)

Primary rectal lymphoma constitutes only 0.1 % of rectal tumours. The diagnosis of primary rectal lymphomas requires that there is a lymphoproliferative disorder confined to the rectum and regional lymph nodes without involvement of abdominal organs, bone marrow and without retroperitoneal or mediastinal lymphadenopathy. Risk factors include AIDS, inflammatory bowel disease and post-transplant status. Rectal lymphoma can present as intraluminal polypoidal mass or diffuse concentric wall thickening. Aneurismal dilatation with minimal or no obstruction is typical with occasional excavating mass with or without fistulisation [31]. It is usually homogenous with intermediate signal on T1 and high signal on T2 with mild to moderate enhancement (Fig. 7). Few recent studies have shown the potential of whole-body DWI in lymphoma for nodal staging, extranodal spread and response assessment. Lymphomatous tissue shows high signal on DWI and low ADC [14]. Differentiating features from adenocarcinoma include preservation of the extramural fat planes, luminal restriction without significant obstruction and thickening of adjacent levator ani [22]. However, it is indistinguishable in a significant number of patients and biopsy is needed for definite diagnosis as the treatment strategies differ considerably. One rare subtype, mantle cell lymphoma commonly presents as multiple polyps called lymphomatous polyposis and can mimic rectal adenomatous polyposis [32] (Fig. 8).

### Stromal tumours

**Fig. 9:**
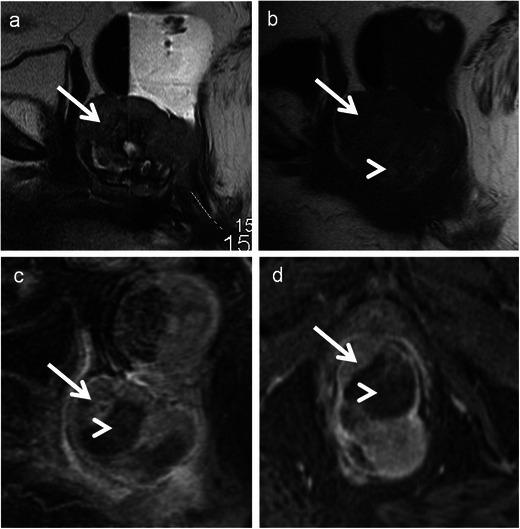
Rectal GIST in a 62-year-old man presenting with pain and rectal bleeding. **a** Sagittal T2-weighted MRI reveals a heterogeneous isointense to hypointense mass (*arrow*) arising from the anterior wall of the rectum and projecting into the rectoprostatic space. **b** Sagittal T1-weighted MRI reveals the lesion to be isointense on T1-weighted MRI with a few areas of hyperintensity (*arrowheads*) representing haemorrhage. **c** Sagittal and **d** axial contrast-enhanced MRI demonstrates intense enhancement (*arrow*) with central areas of non-enhancement (*arrowhead*) representing necrosis

**Fig. 10:**
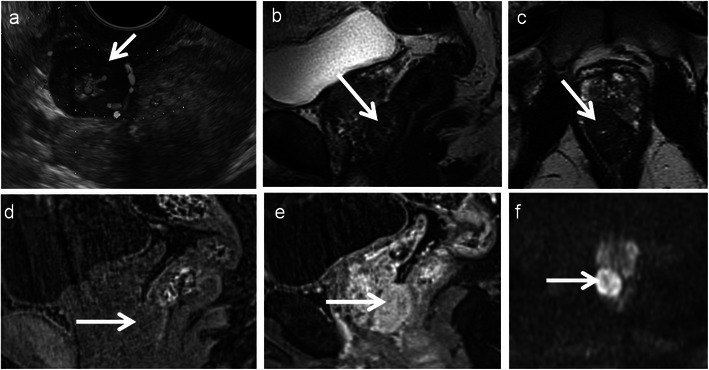
Rectal GIST in a 55-year-old man with a palpable mass on digital rectal examination. **a** Transrectal ultrasound reveals a hypoechoic vascular mass (*arrow*) arising from the rectal wall. **b** Sagittal and **c** axial T2-weighted MRI reveals the lesion (*arrow*) to be heterogeneously hypointense and is arising exophytically from the right anterolateral wall. **d** Sagittal fat-suppressed T1-weighted MRI reveals the lesion (*arrow*) to be intermediate signal intensity and **e** contrast-enhanced T1-weighted MRI reveals intense enhancement (*arrow*). **f** The mass is bright on diffusion-weighted (*b*-1,000) MRI

Gastrointestinal stromal tumours are characterised by unique expression of the c-kit receptor (CD117, tyrosine growth factor receptor), which makes them targets for kit inhibitor therapy. The stomach (60–70 %) and small intestine (20–25 %) are the commonest sites, with the rectum involved in 5–7 % of cases. It is common in males over 50 years. MRI features include well-defined eccentric submucosal masses with an exophytic component showing low signal intensity on T1-weighted imaging, isointense to hyperintense on T2-weighted imaging with marked heterogeneous enhancement. T1 hyperintense areas may suggest haemorrhage and T2 hyperintensity suggest cystic change (Figs. 9 and 10). Large necrotic tumours may cavitate and contain air due to communication with the rectal lumen [33, 34]. The most common mode of spread is peritoneal and haematogenous dissemination to the liver; lymph nodal spread is uncommon. Absence of perirectal adenopathy, despite the large size of the tumour, may suggest GIST instead of adenocarcinoma [35]. Recently DWI has been shown to be potentially capable of offering similar information as PET/CT for diagnosis and treatment response as GISTs show diffusion restriction [15].

### Neuroendocrine tumours

**Fig. 11:**
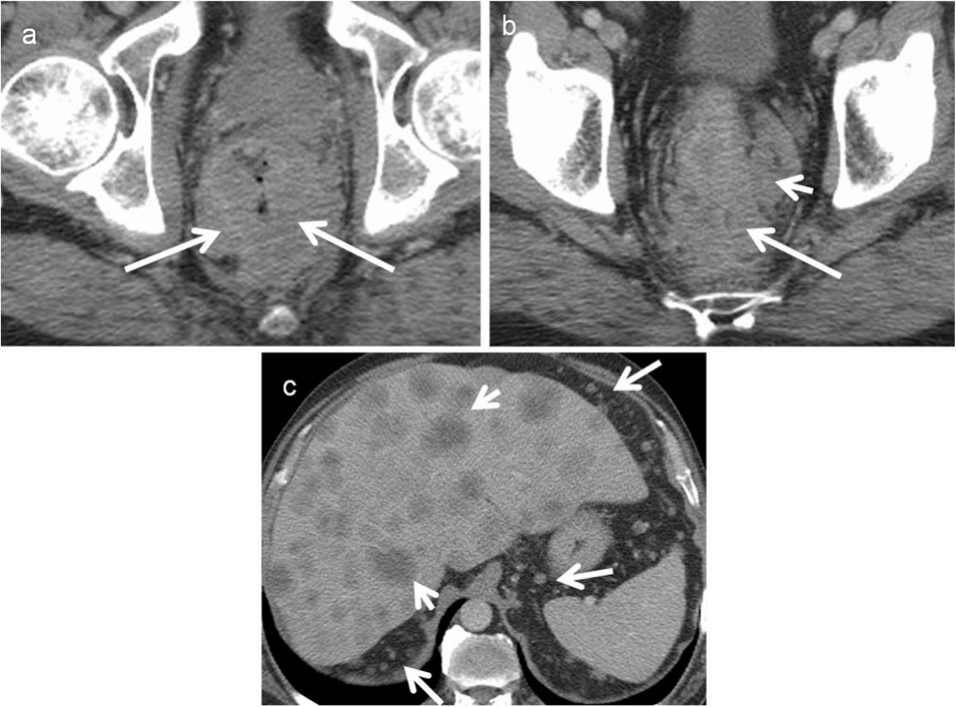
Rectal carcinoid in a 66-year-old woman. **a, b** Axial contrast-enhanced CT reveals lobulated circumferential thickening of the rectal wall (*long arrow*) with perirectal lymph nodes (*short arrow*). **c** Axial contrast-enhanced CT show extensive liver (*short arrow*) and peritoneal (*long arrow*) metastases

Neuroendocrine tumours (NET) are categorised into three groups: well-differentiated neuroendocrine tumour (benign behaviour or uncertain malignant potential; synonymous with carcinoid in gastro-enteric NETs), well-differentiated neuroendocrine carcinoma (low-grade malignancy; synonymous with malignant carcinoid) and poorly differentiated neuroendocrine carcinomas (high-grade malignancy; usually small cell neuroendocrine carcinomas) [36]. Rectum is the second most common gastrointestinal tract carcinoid following small bowel and comprises 27.4 % of all carcinoid of GI tract [37]. Rectal carcinoid is a submucosal tumour with a more indolent course compared with adenocarcinoma. Metastasis occurs in 4–18 % of cases [38]; most commonly to bone, liver and lymph nodes and has better prognosis in rectum compared with other sites. It is located commonly in the mid-rectum on the anterior and lateral wall [39]. On MRI they appear as a solitary, small, submucosal, polypoidal mass, isointense on T1 and isointense to hyperintense on T2 with marked homogenous enhancement [40] (Fig. 11). Management decisions are based on tumour size, necrosis, depth of invasion, mitosis and angiolymphatic invasion. Current treatment recommendations are: <1 cm—endoscopic or trans-anal resection; 1–2 cm, without muscular invasion or lymph node metastasis—wide excision; 2 cm or greater, muscular invasion or lymph node metastasis—radical surgery [22].

### Anorectal melanoma

**Fig. 12:**
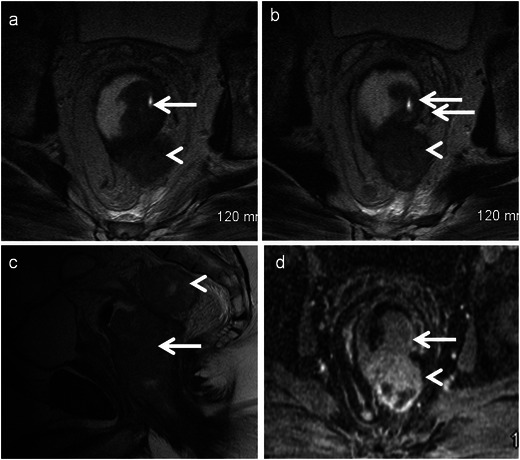
Primary amelanotic anorectal melanoma. **a, b** Axial T2-weighted MRI reveals fungating intraluminal mass (*arrow*) with a large perirectal deposit (*arrowhead*). **c** Sagittal T2-weighted MRI demonstrates the polypoidal mass (*arrow*) expanding the rectum and the large perirectal deposit (*arrowhead*). **d** Axial T1-weighted post contrast MRI reveals heterogeneous enhancement of the mass (*arrow*) and the deposit (*arrowhead*)

Primary melanoma of anorectum is a rare tumour accounting for 0.2–0.3 % of rectal cancers. Typical imaging findings include polypoid intraluminal fungating mass expanding the rectum without obstruction. Bulky perirectal lymphadenopathy is almost always seen (Fig. 12). High T1 signal due to melanin is characteristic but variable as 10–30 % of melanomas are amelanotic type [22]. Sixty percent have already metastasis at the time of initial diagnosis, which includes lymph node, cutaneous and visceral secondaries.

### Kaposi sarcoma

**Fig. 13:**
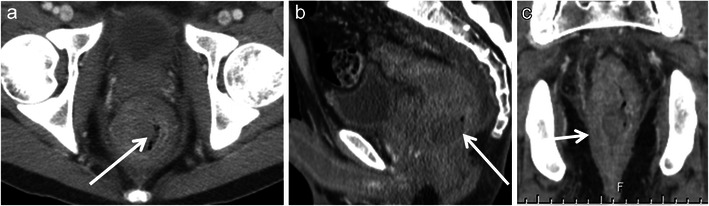
Rectal Kaposi’s sarcoma in a 29-year-old HIV-positive man. **a** Axial, **b** sagittal and **c** coronal CT reveal nodular submucosal mass (*arrow*) in the rectum with rectal wall thickening

Kaposi sarcoma is a low-grade mesenchymal tumour that involves the blood vessels and lymphatics. It primarily affects skin and disseminates in multiple organs usually seen in AIDS or other immunosuppressive states. Digestive tract is involved in more than 50 % of patients, the rectum being second most commonly involved after the duodenum. Commonly present as polypoidal submucosal masses or irregular fold thickening with hyperattenuating disseminated lymphadenopathy in 80 % of patients (Fig. 13) [41]. Bleeding is a dreaded complication and may warrant angiography for diagnosis and treatment.

### Secondary neoplastic involvement

**Fig. 14:**
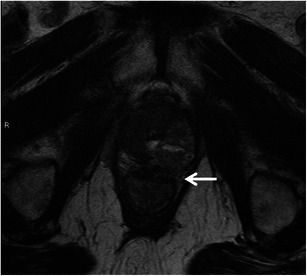
Axial T2-weighted MRI reveals extracapsular extension of prostate cancer with obliteration of the left rectoprostatic angle (*arrow*) and soft tissue mass extending to the anterior serosal surface of the rectum

**Fig. 15:**
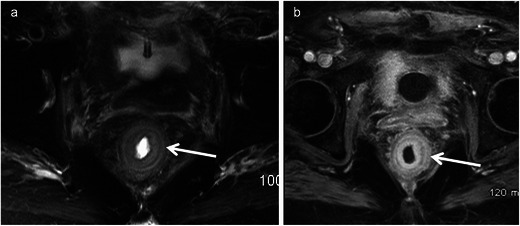
Secondary linitis plastica of rectum from breast cancer. **a** Axial T2-weighted MRI reveals concentric thickening of the rectal wall with a concentric ring pattern likely due to exaggeration of the zonal anatomy. **b** Post-contrast T1-weighted MRI demonstrates similar findings

Secondary neoplastic involvement of rectum is uncommon and it is usually by direct invasion from adjacent organs such as the prostate, urinary bladder, uterus and vagina. Prostatic carcinoma, though, grows close to the anterior wall of the rectum, uncommonly involving the rectum as Denonvillier’s fascia is very firm (Fig. 14) [42]. Rectal metastasis is rare and may present as submucosal mass or as linitis plastica of the rectum. Stomach, breast or prostate primaries can result in linitis plastica of the rectum [43]. Typical imaging features are non-distensible rectum with long segment circumferential thickening and narrowing (Fig. 15). It classically shows a target sign on CT and concentric ring pattern on T2-weighted MRI due to exaggeration of normal zonal anatomy by infiltrative tumour in the submucosa and around the muscle layer [30].

## Miscellaneous lesions

### Deeply infiltrating endometriosis (DIE)

**Fig. 16:**
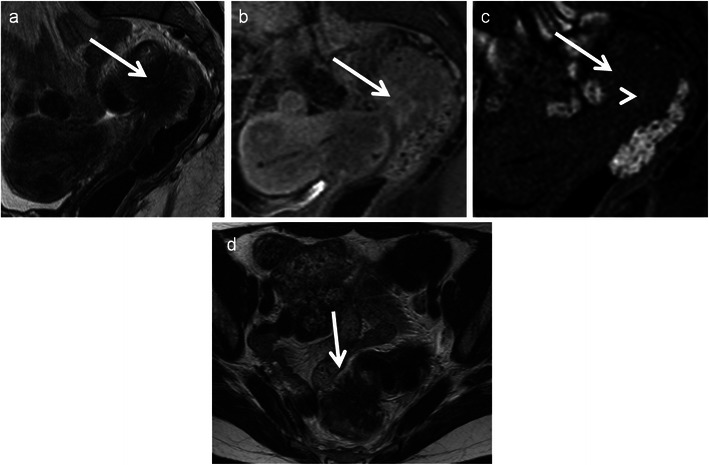
Rectal endometriosis. **a** Sagittal T2-weighted MRI reveals a hypointense spiculated irregular mass (*arrow*) along the anterior surface of the mid rectum. **b** Sagittal T1-weighted MRI reveals this fibrous deposit to be intermediate signal intensity. **c** Sagittal fat-suppressed T1-weighted MRI demonstrates tiny hyperintense foci (*arrowhead*) within the endometriotic deposit (*arrow*). **d** Axial T2-weighted MRI reveals the irregular spiculated hypointense lesion (*arrow*) with a few tiny hyperintense foci

Deeply infiltrating endometriosis is characterised by fibro-muscular hyperplasia that surrounds sparse ectopic endometrial glands. The rectosigmoid is the most common segment of bowel involved. Implants are usually serosal but can erode through subserosal layers and cause thickening and fibrosis of muscularis propria with development of adhesions, bowel strictures and gastrointestinal obstruction. Implants appear as irregular thickening of anterior rectal wall or extramural rectal mass with spiculation and retraction frequently extending to uterosacral ligaments [44, 45, 46]. MRI features are characteristic and include intermediate to high signal on T1 due to blood products and hypointense on T2 due to shading. Shading represents gradual variation in T2 signal due to iron and proteinaceous products from recurrent haemorrhage [47]. Hyperintense foci on T2 may represent ectopic endometrial glands (Fig. 16). However, the disease can also present as fibrous masses that appear to show spiculation and retraction, and hypointense signals on both T1 and T2. Enhancement is variable depending on proportions of inflammatory, glandular and fibrotic tissue [44].

### Lymphogranuloma venerum (LGV)

**Fig. 17:**
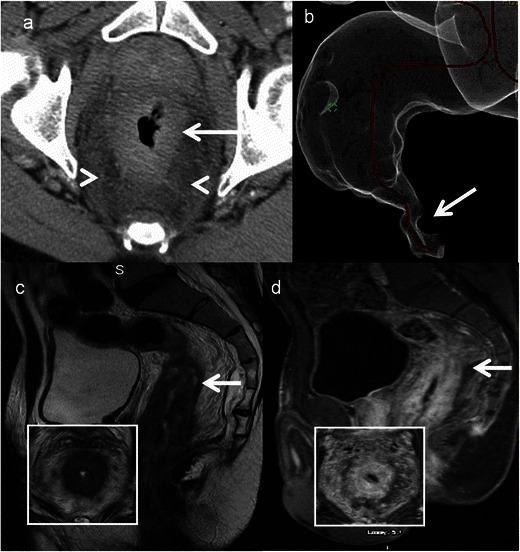
Lymphogranuloma venerum in a 26-year-old HIV-positive man. **a** Axial CT colonography demonstrates diffuse circumferential thickening of the rectum (*arrow*) with inflammatory stranding in the mesorectum (*arrowhead*). **b** Reformatted virtual colonoscopy image demonstrates a smooth stricture (*arrow*) with maintained mucosa. **c** Sagittal T2-weighted MRI and **d** Sagittal contrast-enhanced T1-weighted MRI reveal circumferential thickening (*arrow*) with luminal compromise of the rectum which is hypointense on T2-weighted MRI and shows enhancement post gadolinium administration. Images in *inset* are the corresponding axial MR images showing similar findings

A rare sexually transmitted infection seen in homosexual men, caused by *Chlamydia trachomatis*. It causes ulcerative proctitis mimicking inflammatory bowel disease. Chronic disease leads to strictures and fistulas. Definitive diagnosis is by biopsy and identification of the organism on microbiology [1]. Imaging features are non-specific and include non-specific smooth circumferential thickening of the rectum with adjacent inflammatory stranding. Mucosa is preserved unlike carcinoma (Fig. 17).

### Stercoral colitis

**Fig. 18:**
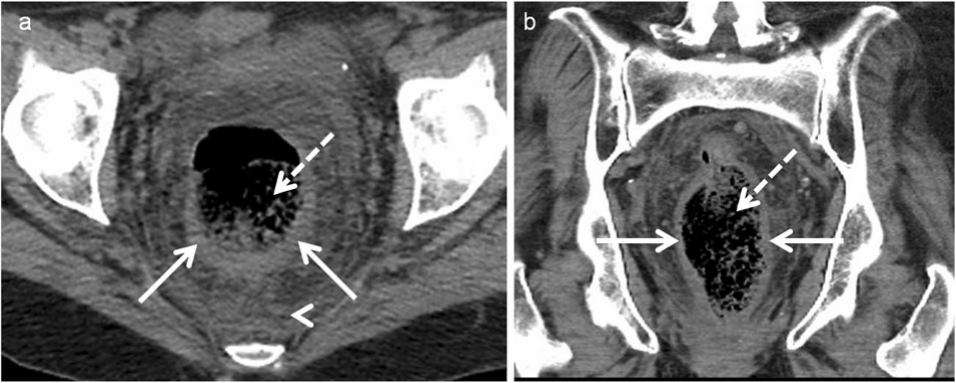
An 81-year-old woman with stercoral colitis. **a** Axial and **b** coronal contrast CT reveal impacted faeces (*dashed arrow*) with thickening of the rectal wall along its posterior aspect (*arrows*) with presacral oedema

Inflammatory colitis related to increased intraluminal pressure from impacted faecal material in the colon. Three most common locations are anterior rectum just proximal to the peritoneal reflection, antimesenteric border of the rectosigmoid and apex of the sigmoid colon [48]. Imaging findings are diagnostic and include faecal impaction with distended affected segment with or without proximal dilatation. Uncomplicated faecal impaction shows a thin wall, whereas focal wall thickening and pericolonic stranding indicate oedema/acute inflammation of stercoral colitis (Fig. 18). Dense mucosa from intramural haemorrhage indicate ischaemia and progresses to mucosal sloughing with perfusion defect indicating infarction [49]. Extra-luminal air and faecalomas protruding through a perforation site indicate perforation. Perforation rent is usually on the antimesenteric border and >1 cm in diameter [50].

### Pseudomyxoma retroperitonei

**Fig. 19:**
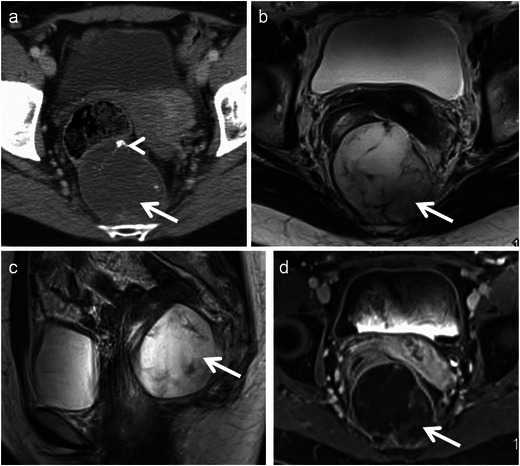
Sub peritoneal pelvic adenomucinosis presenting as slow growing pelvic mass 6 years after surgery for appendiceal mucocele. **a** Axial contrast CT reveals retrorectal multiloculated cystic lesion (*arrow*) with calcification (*arrowhead*). **b** Axial T2-weighted MRI and **c** sagittal T2-weighted MRI reveal a cystic lesion (*arrow*) with multiple septations. **d** Axial contrast T1-weighted MRI reveals peripheral and septal enhancement of the lesion (*arrow*)

Sub-peritoneal pelvic adenomucinosis (pseudomyxoma retroperitonei) is an extra-peritoneal accumulation of mucin, usually following surgery for mucinous appendiceal neoplasms. These are usually indolent and slow growing, and are lined with glandular epithelium and filled with thick, gelatinous material. CT and MR imaging show well-marginated, homogeneous, septated cystic lesions with mural nodules and calcifications (Fig. 19) [51, 52].

## Conclusions

A wide variety of tumours and non-tumorous conditions involve the anorectal region apart from the common adenocarcinoma, inflammatory bowel disease, rectal varices and post-radiation proctitis. A summary of characteristic CT and MRI features are given in Table 1. Cross-sectional imaging, especially MRI, can play an important role in the detection and differentiation of these conditions, although biopsy is often needed for more specific histopathological diagnosis. And for the radiologists, knowledge of the existence of these spectrums of uncommon conditions and their differing imaging appearances is essential for accurate preoperative diagnosis, to differentiate from common rectal adenocarcinoma and thereby to help in appropriate management.

**Table 1:** Summary of characteristic CT and MRI features of various rectal lesions

Lesion		CT features	T1-weighted MRI	T2-weighted MRI	Gadolinium	DWI	ADC	Comments
DEVELOPMENTAL	Tail gut cyst	Multilocular cystic lesion	Hypointense	Hyperintense	Peripheral enhancement	Hyperintense	Hyperintense	Variable T1 and T2 signal due to proteinaceous, mucus and blood products
	Duplication cyst	Unilocular cystic lesion	Hypointense	Hyperintense	Peripheral enhancement	Hyperintense	Hyperintense	Presence of muscle layer and rectal communication
	Epidermoid cyst	Unilocular cystic lesion	Hypointense	Hyperintense	Peripheral enhancement	Hyperintense	Hypointense	Presence T2 hypointense keratin and diffusion restriction
	Dermoid cyst	Unilocular cystic lesion with fat attenuation	Hyperintense	Isointense to hyperintense	Peripheral enhancement	Hyperintense	Hypointense	Hypointense on fat suppression T1 MRI
BENIGN NEOPLASMS	Lipoma	Soft tissue mass with fat attenuation	Hyperintense	Isointense to hyperintense	Mild to variable	Hyperintense	Hypointense	Hypointense on fat suppression T1 MRI
	Leiomyoma	Soft tissue mass	Isointense to hypointense	Isointense to hyperintense	Mild to variable	Variable	Variable	
	Villous adenoma	Carpet or cauliflower like polypoid mass	Hypointense	Hyperintense	Moderate	Hyperintense	Isointense	Enhancement of fibrovascular core
	Schwannoma	Homogenous soft tissue mass	Isointense to hypointense	Hyperintense	Mild homogenous	Isointense to hyperintense	Isointense	
	Cavernous haemangioma	Heterogenous rectal wall thickening with perirectal soft tissue	Isointense to hypointense	Hyperintense	Intense heterogenous	Hyperintense	Isointense to hyperintense	Serpiginious vascular structures and perirectal soft tissue extension
MALIGNANT NEOPLASMS	Mucinous (colloid) carcinoma	Heterogenous hypodense mass	Hypointense	Hyperintense	Heterogenous	Hyperintense	Hypointense	High T2 signal and lace like enhancement
	Lymphoma	Polypoidal mass or diffuse wall thickening	Isointense	Hyperintense	Mild to moderate homogenous	Hyperintense	Hypointense	Aneurismal dilatation and bulky perirectal adenopathy
	Stromal tumours (GIST)	Eccentric soft tissue mass	Hypointense	Isointense to hyperintense	Moderate heterogenous	Hyperintense	Hypointense	Necrosis and haemorrhage seen. Absence of perirectal adenopathy
	Neuro-endocrine tumours	Polypoidal mass	Isointense	Isointense to hyperintense	Marked homogenous	Variable	Variable	
	Anorectal melanoma	Polypoidal fungating mass	Isointense to hyperintense	Isointense to hypointense	Variable	Variable	Variable	Perirectal infiltration and bulky lymph nodes
	Kaposi sarcoma	Polypoidal mass or irregular fold thickening	Isointense to hypointense	Hyperintense	Moderate heterogenous	Variable	Variable	Bulky lymphadenopathy and intratumoral haemorrhage
	Linitis plastica	Long segment circumferential thickening	Hypointense	Concentric ring pattern	Moderate	Variable	Variable	Target sign on CT and concentric ring on MRI
MISCELLANEOUS	Deeply infiltrating endometriosis (DIE)							
	Irregular rectal wall thickening	Isointense to hyperintense	Hypointense	Variable heterogenous	Variable	Variable	Spiculation and retraction of rectal wall	
	Lymphogranuloma venerum (LGV)							
	Smooth circumferential wall thickening	Hypointense	Isointense to hyperintense	Variable	Isointense to hyperintense	Isointense	Smooth strictures with intact mucosa	
	Stercoral colitis	Faecal impaction with wall thickening	Isointense	Isointense to hyperintense	Variable	Hyperintense	Hyperintense	Dense mucosa and perirectal stranding
	Pseudo myxoma retroperitonei	Septated cystic lesions with mural nodules and calcifications	Hypointense	Hyperintense	Peripheral, solid and septal enhancement	Hyperintense	Hyperintense	History of surgery for mucinous neoplasm

*CT* computed tomography, *MRI* magnetic resonance imaging, *DWI* diffusion-weighted imaging, *ADC* apparent diffusion coefficient
